# Abstract of the Proceedings of the Buffalo Medical Association

**Published:** 1862-07

**Authors:** J. F. Miner

**Affiliations:** Secretary


ART. II.—*Abstract of the Proceedings of the Buffalo Medical Association.*

TUESDAY EVENING, May 6, 1861.

Present—Dr. Congar, Vice President, in the Chair; Drs. Gay, Ring, Harvey, Samo, Rochester, Whitney, Cronyn, Miner, Wyckoff.

The Minutes of the last meeting were read, and where Dr. Rochester spoke of the cause of death in the case reported by him, being from blood change, probably spanæmia, corrected to read, probably necræmia, and accepted as published.

*Dr. Congar* read the following report:

Mr. President:—Allow me to present the case of the late B. A. Manchester, Esq., for whom medical advice was given during the period of just six months, viz: from November 3d, 1861, to May 3d, 1862, for diabetis. There were made by his attending physician in conjunction with Prof. Rochester as council, twenty-one chemical examinations of the secretion of the kidneys; one by Prof. Hadley, and four by Prof. A. Clark of New York. Of the twenty-two made in this City, the *range* of the specific gravity of which was between 1024 and 1046; *twelve* were under 1035, and ten were over 1034. By Moore's and Trommer's tests there was ever found *saccharine matter*, varying always in amount with the specific gravity. The quantity of the secretion was daily about thrice that of the healthy average. About the beginning of the fourth month of prescribing for this patient, there were noticed, by the aid of the microscope, *organic* and *fatty granules* in the urine, and also the eyes of the patient showed the "*arcus senilis.*" About this time also, the patient began to feel a sense of aching and weakness, and at times great pain in the region of the kidneys; these sensations never left entirely. About five weeks from this time Dr. A. Clarke discovered *tube-casts*, nearly transparent, in the specimens of urine he examined; and they were ever afterwards present in all the specimens examined. The "rough, dry, and unperspirable skin," common to diabetic patients, was not, at any time, *marked;* but the skin was usually soft and silky, and, much of the time, perspirable. Thirst was *great* some of the time; and, always so *much* as to be more or less annoying. There was always a "*sweet, mawkish taste*" in the mouth. During the last three months the gums were "soft, spongy," and occasionally bleeding; and the lower front teeth were loose from absorption of the alveolar processes and gums. The appetite was usually good, and for about five months bore well an animal diet and bran-bread. The usual "red, raw and dry tongue," of diabetis, appeared but once, and then only during the third week previous to death. During the last month of life *only* did the usual "stomachic uneasiness" show itself. "*Constipation of the bowels*" was particularly obstinate during the last three months. For five weeks before death there came on "a severe, tickling cough, pain in different parts of the chest, always evanescent, and a free expectoration of mucus." In the treatment, *Camplin's plan was thoroughly tried.* The whole treatment may, however, be comprised in a *discriminate use* of "animal diet," "opiates," "sudorifics," "alkalies" and "tonics." With these were combined as skillfully as possible, considering the business condition and habits of the patient, and the season of the year, *rest, air,* and *exercise.* And yet from the commencement of treatment, the check of decline was very slight; in fact, during the whole time of his medical guidance, the decline was rapid, and at last, so rapid, as to resemble those dying from other collequative discharges, as cholera, &c.

*Dr. Wyckoff* inquired if Camplin's plan of treatment had ever been attended by success, or if patients were generally greatly and permanently benefited by it, or any other medication.

*Dr. Rochester* had known some cases benefited. Capt. Potter, Dr. White's patient, thought himself well, at least for a time; does not know how it may be at present. Mr. E. S. Rich gave Mr. Manchester Dr. Camplin's book upon the subject. His plan consists in tonics, stimulants, strychnine, and especially *bran bread;* the process for making which is fully given in the book, and is the principal point in the treatment. The bran is prepared by washing so as to leave no starch, and is advised as the only food.

*Dr. Wyckoff* inquired if the use of cider was known to be attended by benefit.

*Dr. Rochester* had known it tried often; Mr. Manchester had tried it; had not known it to be useful.

*Dr. Harvey* knew a man who had been benefited by Petroleum Oil. It relieved him very much.

*Dr. Rochester* spoke of the great importance of correct diagnosis, at least in arriving at conclusions as to the benefit from different modes of treatment. Remarked also upon the frequency of *supposed* cases, when in fact, the urine contained no sugar. Incontinance of urine in old people, or constant desire to void it, was often *called* diabetis, especially by patients, encouraged to do so by physicians, oftentimes.

*Dr. Cronyn* gave brief account of post mortem examination of a Mr. Miller, supposed by some to have diabetis. Kidneys greatly atrophied. Supra renal capsules obliterated or suppurated; arcus senilis well marked. There was fatty degeneration.

*Dr. Ring* had treated one case of diabetis from July to October. Kreosote had diminished the quantity of water and benefitted the patient very much; gave no other medicine; did not know the patient to be still alive. The urine and the amount of sugar were both diminished under the use of kreosote, and also a peculiar odor, which was present.

*Dr. Rochester* had never known a well authenticated case of recovery. Dr. Frick speaks of the value of kreosote and also of strychnia in some valuable papers upon the subject, and says diminished quantity of urine is no evidence of improvement, that the sugar is often found in the fæces and other secretions.

*Dr. Wyckoff* confined his patient to bran bread, tonics, quinine and iron. He also gave kreosote, which diminished the quantity of urine, but produced no benefit. He had omitted medication.

*Dr. Gay* read the following letter:

"BYRON, May 14, 1862.

Dear Doctor:—As you were acquainted with Mr. Atkins of this place, you may be interested in a brief notice of the post mortem examination which I was permitted to make. Upon opening the thorax I found the *right* lung entirely wanting, and no indication that one had ever existed. It was probably destroyed about twenty years since by disease which he then suffered from. The heart was lying in the space once occupied by the lung, considerably higher than its natural position, and thus easily accounted for the pulsation which was accidentally discovered in the right side, about sixteen years since, and by some physicians thought to be an aneurism. The organ was small, and its walls considerably attenuated. The tricuspid and aortic valves were imperfect, being contracted and cartilaginous. The left lung was emphysematous, and adherent throughout its entire surface. The apex was filled with small abscesses of tubercular matter and some chalky deposits. Upon looking into the abdomen we discovered the right kidney to have undergone complete organic degeneration. The gland was shorter and thicker than natural, with the larger curve scolloped. A semi-transparent flesh-colored membrane was filled with a white substance resembling thick paint, or lardaceous degeneration, more properly speaking. The bladder, which for many years had been very irritable, was small, with a portion of it thickened to half an inch and containing pus in its walls. In it were found a number of small, soft concretions which occasionally interrupted the passage of urine.

Mr. A. has been troubled more or less with a cough for many years, but has been confined to his house for a few months only. The utmost care, and prudence have enabled him to live many years under the most unfavorable circumstances.

Yours in haste, R. WILLIAMS."

Dr. Shaw of Buffalo, was proposed for membership.

Voted to adjourn to Tuesday, July 1st, 1862.

J. F. MINER, Secretary.

---

DATURA STRAMONIUM.—The active principle of this plant resides chiefly in the seeds. The researches of M. Hirtz prove that there exists a great pharmaco-dynamic and therapeutical similarity between datura stramonium and belladonna. The therapeutical effect of stramonium in asthma was most remarkable when used in the form of segars; but when employed internally in the same disease it was not less efficacious, although less rapid in its action.

